# Contralateral versus ipsilateral vaccine boosting for COVID-19: considering the broader scientific landscape

**DOI:** 10.1172/JCI179149

**Published:** 2024-03-15

**Authors:** Paul Goepfert

**Affiliations:** Department of Medicine, The University of Alabama at Birmingham Marnix E. Heersink School of Medicine, Birmingham, Alabama, USA.

## Abstract

In the relentless battle against the COVID-19 pandemic, the deployment of mRNA vaccines has stood out as a beacon of hope. The successes of Pfizer-BioNTech NT162b2 and Moderna mRNA-1273 vaccines have been remarkable, marking a revolutionary advancement in the field of vaccinology. Despite their rapid development and impressive efficacy, challenges have emerged, particularly concerning the waning immune response over time and the evolving landscape of SARS-CoV-2 variants. The study published in this issue of *JCI* by Fazli et al. introduces an approach to potentially enhancing the immune responses generated by COVID-19 mRNA vaccines. The study meticulously examines the outcomes of nearly 1,000 participants who received one or two booster doses with the Pfizer-BioNTech NT162b2 vaccine either ipsilaterally or contralaterally in relation to the initial vaccine dose. Intriguingly, those who received the booster contralaterally exhibited a heightened antibody response that was particularly noteworthy in the later time points after boost.

## mRNA COVID-19 vaccination successes and limitations

The advent of mRNA technology in vaccine development has been transformative, allowing scientists to respond rapidly to the urgent global need for effective COVID-19 vaccines (1). The process involves decoding the genetic sequence of the virus and designing mRNA sequences that encode the viral spike protein. This innovative approach instructs cells to produce the viral protein, triggering a robust immune response that includes all subsets of the adaptive immune response.

Both Pfizer-BioNTech NT162b2 and Moderna mRNA-1273 vaccines demonstrated remarkable efficacy and safety in large-scale clinical trials (2, 3). Their accelerated development and approval represented a paradigm shift in vaccine time lines, showcasing the potential of mRNA technology to swiftly address emerging infectious threats. The success of these vaccines has not only played a crucial role in mitigating the COVID-19 pandemic, saving millions of lives, but has also set a precedent for future vaccine development (4).

While the advantages of mRNA vaccines include rapid development and robust immune response induction, challenges have surfaced. One notable concern is the potential for waning immunity over time (5, 6). Studies have indicated a gradual decrease in the antibody response several months after vaccination, raising questions about the long-term effectiveness and the necessity for booster shots to sustain protection. Additionally, the evolution of SARS-CoV-2 has introduced new variants with changes in the spike protein, potentially affecting the efficacy of existing vaccines (7). The ability of mRNA vaccines to adapt quickly to new viral strains is advantageous, but it necessitates continuous research and vaccine adjustments, posing logistical and regulatory challenges.

Addressing these challenges is imperative for maintaining the effectiveness of COVID-19 vaccination strategies and staying ahead of the virus’s evolving nature. Strategies for enhancing the durability of immune responses become paramount, particularly as global populations may be hesitant about additional vaccines (8).

## Contralateral mRNA COVID-19 boosting improves antibody magnitude

**Figure 1:**
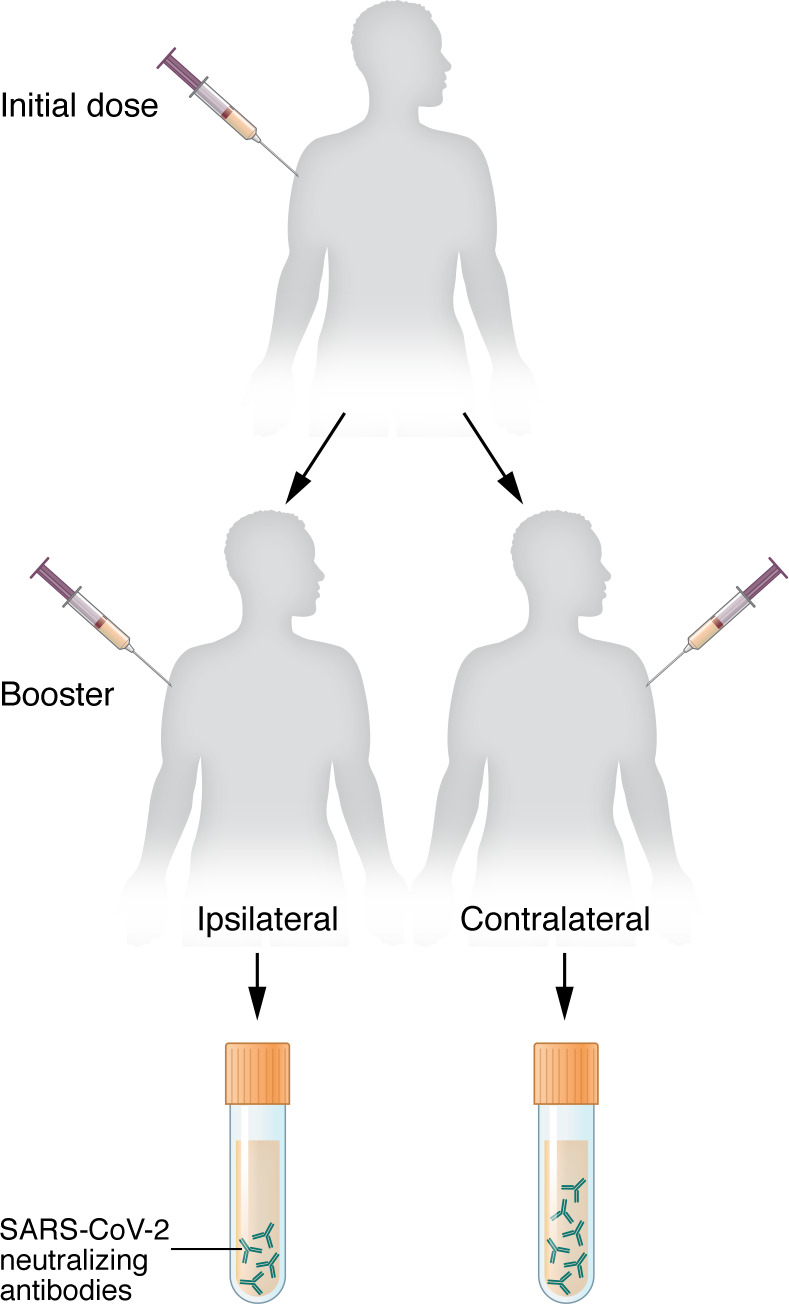
Contralateral COVID-19 boost enhances antibody responses. Participants in Fazli et al. (9) received the first dose of the Pfizer-BioNTech NT162b2 vaccine, then were randomized to receive one or two boosters either ipsilaterally or contralaterally with respect to the first vaccine. SARS-CoV-2 neutralizing antibodies were analyzed after boosting. Participants who received contralateral injections showed higher neutralizing titers.

In the pursuit of improving COVID-19 vaccine immune responses, Fazli et al.’s study examined the impact of administering booster doses in the same or contralateral arms (9) (Figure 1). In contrast to some recent findings (10), the current study reports that boosting with Pfizer-BioNTech NT162b2 in those previously primed with the initial vaccine resulted in a higher magnitude of antibody responses. This difference was most pronounced at the last time point analyzed, approximately five months after the third vaccination. Notably, the study focused on neutralizing antibody responses, including those against the Omicron variant (B.1.1.529), revealing enhanced antibodies with contralateral boosts. Higher antibody levels also correlate with improved crossneutralization of variant strains (11), addressing a crucial concern in the face of evolving viral threats.

The study’s robust methodology, encompassing a large cohort and thorough participant enrollment and demographics analysis, strengthens the reliability of its findings. This work contributes valuable insights into the optimization of vaccine administration, emphasizing the relevance of the injection site for booster doses. The potential implications of these results for vaccine deployment strategies could influence vaccination guidelines and shape future research on optimizing immune responses through strategic injection-site choices.

However, the authors appropriately acknowledge the need for verification in larger cohorts before making recommendations for current practices. It is noteworthy that a recently published study by Ziegler et al. presented contradictory findings, demonstrating improved antibody responses with ipsilateral boosting of COVID-19 vaccination (10). Furthermore, a nonrandomized, retrospective study in a large number of vaccinees demonstrated that ipsilateral COVID-19 second-dose administration resulted in reduced PCR-confirmed COVID-19 infections (12). The differences among these studies, such as the design of study, the number of participants, and the timing of analyses, underscore the complexity of immune responses and highlight the necessity for further investigation.

The divergence in findings between these studies adds complexity to the interpretation of optimal vaccination strategies. While Fazli et al.’s (9) study indicates the superiority of contralateral boosts, it is crucial to consider the broader scientific landscape. In addition to the aforementioned clinical studies (10, 12), at least two well-designed studies using animal models demonstrated the superiority of ipsilateral boosts, further complicating the picture (13, 14). The need for additional preclinical and clinical studies to confirm and reconcile these findings becomes apparent.

## Future directions

As is often the case with well-executed studies, Fazli et al.’s findings not only provide direction for further research, but also raise several important questions (9). Exploring the breadth of antibody responses based on contralateral or ipsilateral boosting is crucial, as is understanding differences in B and T cell responses. These insights could further inform strategies for optimizing immune responses against SARS-CoV-2.

The impact of contralateral versus ipsilateral boosting on various vaccine types and adjuvants warrants investigation. Understanding whether these findings extend beyond mRNA vaccines to other vaccine platforms is essential for developing comprehensive vaccination strategies. Additionally, exploring the influence of boost timing and the use of heterologous strain vaccines on antibody responses adds another layer of complexity to the research agenda.

Mechanistic understanding of the observed effects is useful for refining future vaccines and instilling confidence in the findings. Unraveling the intricate interplay between injection-site choices and immune responses could pave the way for more tailored and effective vaccination strategies. Collaborative efforts across disciplines and continued vigilance in monitoring the evolving landscape of SARS-CoV-2 will be crucial for staying ahead of the virus and optimizing vaccination approaches.

In conclusion, the study by Fazli et al. provides a valuable contribution to the ongoing discourse on COVID-19 vaccination strategies (9). The contrasting results regarding contralateral versus ipsilateral boosting strategies in different papers to date underscore the complexity of immune responses and the need for comprehensive investigations. As the world grapples with ongoing vaccination efforts and potential future threats, research endeavors exploring innovative approaches to enhancing vaccine responses remain an important scientific issue.

## Version 1 03/15/2024

Electronic publication
